# The CAM-ICU-7 and ICDSC as measures of delirium severity in critically ill adult patients

**DOI:** 10.1371/journal.pone.0242378

**Published:** 2020-11-16

**Authors:** Karla D. Krewulak, Brianna K. Rosgen, E. W. Ely, Henry T. Stelfox, Kirsten M. Fiest

**Affiliations:** 1 Department of Critical Care Medicine, Cumming School of Medicine, University of Calgary, Calgary, Canada; 2 Department of Community Health Sciences and O’Brien Institute for Public Health, Cumming School of Medicine, University of Calgary, Calgary, AB, Canada; 3 Tennessee Valley Veteran’s Affairs Geriatric Research Education Clinical Center (VA GRECC), Critical Illness, Brain Dysfunction, and Survivorship (CIBS) Center, Vanderbilt University Medical Center, Nashville, Tennessee, United States of America; 4 Department of Critical Care Medicine, Alberta Health Services, Calgary, Alberta, Canada; 5 Department of Psychiatry & Hotchkiss Brain Institute, Cumming School of Medicine, University of Calgary, Calgary, AB, Canada; BronxCare Health System, Affiliated with Icahn School of Medicine at Mount Sinai, UNITED STATES

## Abstract

**Background:**

In clinical practice, a dichotomous approach to delirium identification may no longer be relevant when existing delirium screening tools measure a range of scores. The objective of this study was to compare the Confusion Assessment Method for the Intensive Care Unit 7-item (CAM-ICU-7) and the Intensive Care Delirium Screening Checklist (ICDSC) as measures of the spectrum of delirium severity in critically ill adult patients.

**Methods:**

In this cross-sectional study, 218 patients underwent 641 paired assessments by bedside nurses (ICDSC, as per usual care) and trained research assistants (CAM-ICU-7). Correlation between the CAM-ICU-7 and ICDSC scores was evaluated. Logistic regression was used to explore associations between CAM-ICU-7 or ICDSC score and length of ICU stay and mechanical ventilation (receipt, ≥96 hours).

**Results:**

Delirium prevalence evaluated by the CAM-ICU-7 and ICDSC were 46.3% (95% CI:39.7–53.0) and 34.4% (95% CI:28.3–41.0). Prevalence of less than clinical threshold symptoms of delirium evaluated by the CAM-ICU-7 (score: 1–2) and ICDSC (score: 1–3) were 30.3% (95%CI:24.5–36.7) and 50.9% (95%CI:44.3–57.6). The CAM-ICU-7 and ICDSC had significant positive correlation (0.58, p<0.001). Agreement between the tools as measures of delirium was moderate (kappa = 0.51) and as measures of less than clinical threshold symptoms of delirium was fair (kappa = 0.21). Less than clinical threshold symptoms of delirium identified by the ICDSC, not CAM-ICU-7, were associated with prolonged length of ICU stay (≥7 days) in patients <65 years of age [Odds Ratio (OR) 9.2, 95% CI:2.5–34.0] and mechanical ventilation (receipt: OR 2.8, 95% CI:1.3–6.4; ≥96 hours: OR 6.6, 95% CI:1.9–22.9), when compared to patients with no delirium.

**Conclusions:**

The CAM-ICU-7 and ICDSC are measures of the spectrum of delirium severity that are closely correlated. Less than clinical threshold symptoms of delirium measure by the ICDSC is a better predictor of outcomes, when compared with the CAM-ICU-7.

## Introduction

Delirium is a serious neuropsychiatric syndrome that affects nearly 50% of critically ill patients while in the Intensive Care Unit (ICU) [1–3]. To date, delirium has been conceived as a dichotomous circumstance wherein delirium exists, or it does not. Delirium screening tools such as the Confusion Assessment Method for the Intensive Care Unit (CAM-ICU) [4] and the Intensive Care Delirium Screening Checklist (ICDSC) [5,6] allow for measurement of a range of delirium scores; a dichotomous approach to delirium measurement may no longer be relevant in clinical practice. Instead, the measurement of the spectrum of delirium severity should be considered.

Previous studies compare the CAM-ICU and ICDSC as measures of delirium presence or absence and with high agreement [7–11]. The CAM-ICU and ICDSC have different approaches. The CAM-ICU uses an algorithm where three features of delirium (i.e., fluctuating course/sudden onset and inattention and either altered level of consciousness or disorganized thinking) must be present for the CAM-ICU to be positive [12,13]. The ICDSC is score-based (range 0–8) where the ICDSC is positive when any four (or more) symptoms of delirium are present (i.e., altered level of consciousness, inattention, disorientation, hallucinations or delusions, psychomotor activity, inappropriate speech or mood, sleep disturbance or fluctuation of symptoms) [6]. Recently, a score-based version of the CAM-ICU (CAM-ICU-7) was developed, with each feature of delirium being assigned a score based on the severity of the disruption [4]. Recent studies suggest measurement of the spectrum of delirium severity, which is possible with score-based delirium detection, in clinical practice may be an important means to identify the earliest onset of symptoms of delirium, target delirium prevention and management strategies, track the effectiveness of these strategies and monitor outcomes for patients with delirium [14,15]. Patients with less than clinical threshold symptoms of delirium (commonly referred to as subsyndromal delirium) could benefit from early nonpharmacological interventions: up to 40% cases of delirium can be prevented [16] or may not progress to clinical delirium [17].

The aim of the present study was to compare the CAM-ICU-7 and the ICDSC as measurements of the spectrum of delirium severity in critically ill patients and their association with short term outcomes of critically ill adults.

## Materials and methods

### Study setting

**Fig 1:**
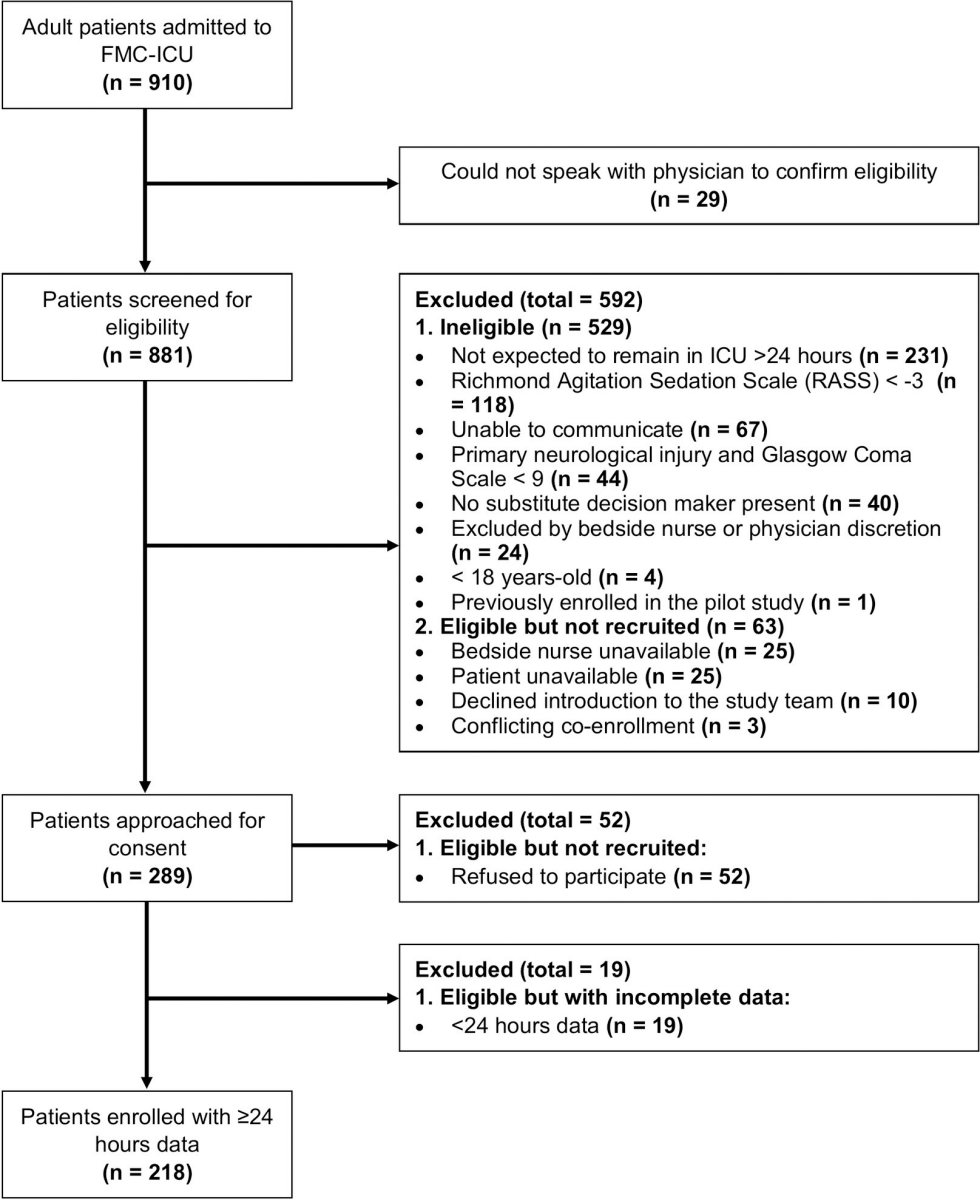
Patient participant flow diagram.

Patients in this cross-sectional study were recruited at a 28-bed, medical-surgical ICU at Foothills Medical Center (FMC ICU) in Calgary, Canada (catchment population: 1.8 million) between November 2017 and March 2019. FMC ICU utilizes all components of the ABCDEF bundle, including routine delirium assessment using the ICDSC once per shift. Metrics for each component are recorded on a bedside critical care clinical information system (eCritical) and are regularly audited to ensure ABCDEF bundle compliance. We followed the Strengthening the Reporting of Observational Studies in Epidemiology (STROBE) guidelines for cross-sectional studies [18] (Fig 1). We included adults (≥18 years of age) with no primary direct brain injury (pre-existing neurological comorbidities [e.g., epilepsy, multiple sclerosis, dementia] was not an exclusion criteria), who could provide consent or surrogate consent, could communicate with the study team (i.e., understand English, no hearing impairment) and were expected to remain in the ICU for at least 24 hours. Patients with a Richmond Agitation-Sedation Scale (RASS) of -4 or -5 or Glasgow Comma Scale of ≤ 9 [19] were excluded. The study was approved by the Conjoint Health Research Ethics Board at the University of Calgary (Reference number: REB16-2060).

### Sampling & recruitment

All consecutive, eligible patients admitted to the FMC ICU or their surrogate decision makers were asked by the bedside registered nurse if a research assistant could approach them to discuss a research opportunity. The patient’s capacity to provide written, informed consent was decided by the bedside registered nurse. If the patient (or surrogate decision maker) agreed, informed consent was sought, and the patient was enrolled in the study. If a patient regained capacity, consent was sought.

### Delirium assessments

The RASS was used to assess a patients’ level of sedation or agitation [20]. The RASS is a tool with excellent interrater reliability (k = 0.91, 95% confidence interval (CI) = 0.86–0.95) [20]. Patients with a RASS score of -4 or -5 were not eligible for delirium assessments and, as such, were marked as a missed delirium assessment for that day. Delirium was assessed twice daily in all patients using the CAM-ICU-7 (at 11:00 and 16:00) and ICDSC (at 06:00 and 18:00) for up to five days during their ICU stay. The CAM-ICU-7 and RASS were performed by trained research assistants (KDK, BKR), who were blinded to all bedside registered nurse’s delirium and RASS assessments conducted during the five days of data collection. The ICDSC and RASS assessments were performed by trained bedside registered nurses who were also blinded to the study team’s delirium and RASS assessments conducted within the first 12 hours of ICU admission.

The CAM-ICU-7 has a 7-point rating scale (range: 0–7 points) that is derived from the CAM-ICU and RASS assessments [4]. The CAM-ICU-7 evaluates the presence of acute onset or fluctuating course (score of 0 or 1), inattention (score ranges from 0–2), altered level of consciousness (score ranges from 0–2, based on if RASS is anything other than alert and calm [zero]), and disorganized thinking (score ranges from 0–2). A patient is considered to have no delirium with a score of 0–2, mild to moderate delirium with a score of 3–5 and severe delirium with a score of 6–7 [4]. The CAM-ICU-7 has high internal consistency (Cronbach's alpha = 0.85) and good correlation with another delirium severity scale: Delirium Rating Scale-Revised-98 (DRS-R-98) (correlation coefficient = 0.64) [4]. The CAM-ICU has a pooled sensitivity among nine studies of 80.0% (95% CI: 77.1–82.6%) and pooled specificity of 95.9% (95% CI: 94.8–96.8%) [10].

The ICDSC is an 8-item delirium screening instrument (range: 0–8 points) that evaluates a patient’s level of consciousness, inattention, disorientation, hallucinations or delusions, psychomotor activity, inappropriate speech or mood, sleep disturbance and fluctuation of symptoms [5,6]. Each item is rated based on the patient’s behaviour over the previous 12 hours. The ICDSC has high internal consistency (Cronbach's alpha = 0.84) [21]. The ICDSC has a pooled sensitivity among four studies of 75% (95%CI: 65.3–81.5%) and pooled specificity of 81.9% (95%CI: 76.7 to 86.4%) [10].

**Fig 2:**
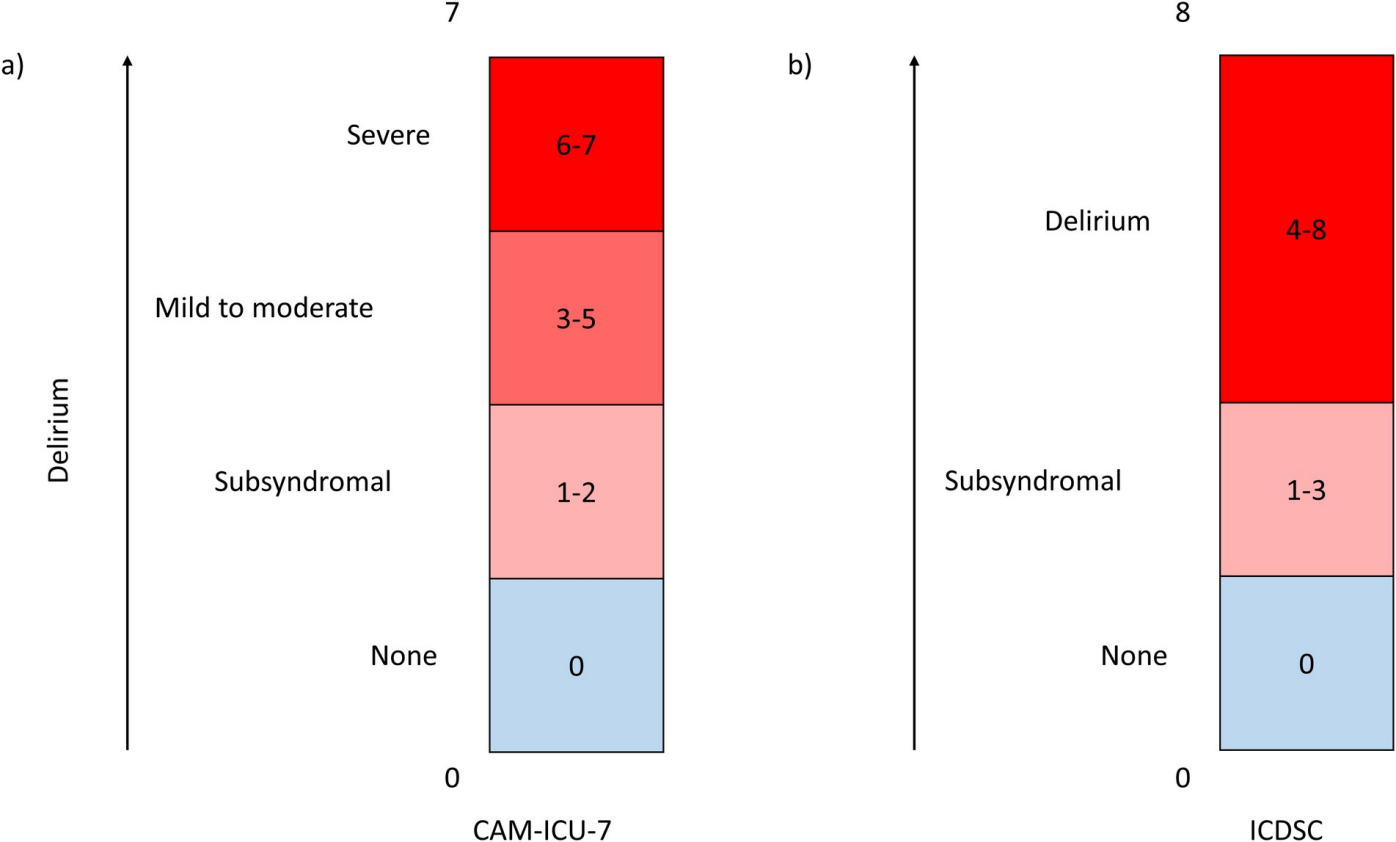
Visual representation of the a) CAM-ICU-7 and b) ICDSC as measurements of the spectrum of delirium severity. If a patient exhibits less than clinical symptoms of delirium (i.e., negative CAM-ICU-7 and one of the CAM-ICU features [acute change, fluctuating course, altered level of consciousness, disorganized thinking] or 1–3 items on the ICDSC [altered level of consciousness, inattention, disorientation, hallucination/delusion/psychosis, psychomotor agitation or retardation, inappropriate mood or speech, sleep wake cycle, fluctuations), less than clinical threshold symptoms of delirium (commonly referred to as subsyndromal delirium) is present (light pink box). A patient is considered to screen positive for delirium if their CAM-ICU-7 score is greater than or equal to three or their ICDSC score is greater than or equal to four (red box).

A comparison between the CAM-ICU-7 and ICDSC as a measurement of the spectrum of delirium severity is demonstrated in Fig 2. To assess the spectrum of delirium severity, less than clinical threshold symptoms of delirium were considered present if a CAM-ICU-7 assessment was negative, but at least one CAM-ICU feature was present (i.e. acute change or fluctuating course, inattention, altered level of consciousness or disorganized thinking) also represented by a CAM-ICU-7 score of 1–2 [22]. For the ICDSC, a patient was considered to have no delirium with a score of 0, less than clinical threshold symptoms of delirium with a score of 1–3 and clinical delirium with a score of 4–8 [5,6].

### Other data and clinical outcomes

The patient (or surrogate) completed a demographics questionnaire that included age, education, ethnicity, gender and sex). Clinical characteristics such as severity of illness (Acute Physiology and Chronic Health Evaluation [APACHE] II score upon ICU admission) and organ failure (Sequential Organ Failure Assessment [SOFA] score upon ICU admission) were obtained from eCritical, which is validated for use in research [23]. We identified short term outcomes that were associated with delirium [24] and may be correlated with CAM-ICU-7 or ICDSC scores to assess predictive validity. These short-term outcomes were collected from electronic medical records and included receipt/length of invasive mechanical ventilation during the ICU stay, length of ICU stay and ICU mortality.

### Statistical analyses

Descriptive statistics were examined for all study variables. Continuous variables with a normal distribution were presented as mean ± standard deviation [SD]. Correlation between the CAM-ICU-7 and ICDSC scores were estimated by Pearson’s correlation coefficient, wherein a Pearson r value of 0.10, 0.30, and 0.50 was interpreted as small, medium, and large effect sizes, respectively [25]. Kappa was calculated as a measure of agreement between the meaning of a CAM-ICU-7 or ICDSC score (i.e., less than clinical threshold symptoms of delirium, delirium), wherein agreement was interpreted as fair (0.21–0.40), moderate (0.41–0.60), substantial (0.61–0.80) and almost perfect (0.81–1.00) [26]. Variables considered potential effect modifiers or confounders were identified *a priori*, and included age, sex and severity of illness (APACHE-II score upon ICU admission). Race was not included as a potential effect modifier or confounder because ICU literature reports no association between race and delirium [27,28]. Logistic regression analysis was used to assess the relationship of prolonged length of ICU stay (≥7 days), receipt of invasive mechanical ventilation (ever/never), invasive mechanical ventilation ≥96 hours [29,30] and ICU mortality with delirium severity. Box plots were used to show median and quartiles of length of ICU stay and length of mechanical ventilation with respect to CAM-ICU-7 and ICDSC score categories. For all analyses, one delirium measure per patient (the most severe CAM-ICU-7 or ICDSC score) was used. All analyses were performed using Stata, version 14.0 (StataCorp, College Station, TX, USA).

## Results

From November 2017 to March 2019, 910 patients were screened, 356 patients were eligible and 218 patients were enrolled, with a participation rate of 61.2% (218/356) (Fig 1). The majority of enrolment was completed by patient consent (55.5%, 121/218). The majority of participants were male (59.2%, 129/218), with a mean age of 58.8 years (SD 15.5) (Table 1). Delirium was identified in 46.3% (95% CI: 39.7–53.0) of patients with the CAM-ICU-7 and 34.4% (95% CI: 28.3–41.0) of patients with the ICDSC. The proportion of female sex and gender identity were the same and, as such, only sex will be considered in the analysis.

**Table 1:** Characteristics of the study sample (n = 218).

CAM-ICU-7	No delirium (n = 51)		Score 1–2 (n = 66)		Score 3–5 (Delirium) (n = 61)		Score 6–7 (Delirium) (n = 40)
Age, mean (SD), years	55.0 (17.2)		55.3 (15.7)		56.4 (15.3)		62.0 (12.1)
Female sex, n (%)*	22 (43.14)		34 (51.5)		19 (31.1)		14 (35.0)
APACHE-II score, median (IQR)	18.0 (12.0)		18.0 (10.0)		21.0 (10.0)		21.5 (11.0)
SOFA score, median (IQR)	5.0 (5.0)		6.0 (4.0)		7.0 (5.0)		8.0 (3.5)
Invasive mechanical ventilation, n (%)	33 (64.7)		47 (71.2)		45 (73.8)		38 (95)
Invasive mechanical ventilation duration, median (IQR), hours	63.1 (110.2)		74.8 (132.0)		165.1 (166.5)		202.4 (198.6)
ICU length of stay, median (IQR), days	6.7 (5.5)		6.2 (6.0)		10.8 (9.6)		16.2 (12.8)
Hospital length of stay, median (IQR), days	16.5 (17.4)		23.9 (47.6)		27.7 (32.1)		35.9 (37.7)
ICU mortality, n	2		2		0		3
Hospital mortality, n	2		12		8		8
Admitting diagnosis, n (%):							
Medical	23 (45.1)		29 (59.1)		34 (55.7)		16 (40.0)
Neurologic	8 (15.7)		8 (12.1)		14 (22.9)		11 (27.5)
Trauma	10 (19.6)		8 (12.1)		6 (9.8)		8 (20.0)
Surgical	10 (19.6)		11 (16.7)		4 (6.6)		5 (12.5)
Education, n (%):							
More than a high school	36 (70.6)		47 (71.2)		45 (73.8)		25 (62.5)
High school or less	15 (29.4)		17 (25.8)		12 (19.7)		13 (32.5)
Other	0 (0)		2 (3.0)		3 (4.9)		1 (2.5)
Not provided	0 (0)		0 (0)		1 (1.6)		1 (2.5)
^a^^,^^b^Ethnicity/race, n(%)							
Asian or Pacific Islander	0 (0)		5 (7.6)		8 (13.1)		4 (10.0)
Black/African American)	0 (0)		3 (4.5)		1 (1.6)		0 (0)
Caucasian/White	9 (17.6)		8 (12.1)		5 (8.2)		5 (12.5)
European	21 (41.2)		19 (28.8)		16 (26.2)		12 (30.0)
Hispanic	0 (0)		0 (0)		0 (0)		1 (2.5)
Indigenous	2 (3.9)		5 (7.6)		2 (3.3)		0 (0)
Middle Eastern	1 (2.0)		2 (3.0)		0 (0)		0 (0)
North American	15 (29.4)		21 (31.8)		24 (39.3)		13 (32.5)
South American	1 (2.0)		0 (0)		0 (0)		0 (0)
**ICDSC**		**No delirium (n = 32)**		**Score 1–3 (n = 111)**		**Score 4–8 (Delirium) (n = 75)**	
Age, mean (SD), years		53.0 (20.5)		56.7 (14.5)		58.6 (14.3)	
Female sex, n (%)		15 (46.9)		49 (44.1)		25 (33.3)	
APACHE-II score, median (IQR)		15.5 (12)		19 (12)		21 (9)	
SOFA score, median (IQR)		5.0 (5.5)		6.0 (5.0)		7.0 (4.0)	
Invasive mechanical ventilation, n (%)		16 (50)		82 (73.9)		65 (86.7)	
Invasive mechanical ventilation duration, median (IQR), hours		29.1 (40.2)		115.5 (192.1)		168.7 (168.9)	
ICU length of stay, median (IQR), days		4.8 (4.6)		8.5 (10.3)		12.4 (10.8)	
Hospital length of stay, median (IQR), days		13.8 (32.8)		22.4 (35.2)		29.8 (42.2)	
ICU mortality, n		0		3		4	
Hospital mortality, n		1		13		16	
Admitting diagnosis, n (%):							
Medical		22 (68.7)		59 (53.1)		31 (41.3)	
Neurologic		5 (15.6)		13 (11.7)		23 (30.7)	
Trauma		3 (9.3)		15 (13.5)		12 (16.0)	
Surgical		2 (6.2)		24 (21.6)		9 (12.0)	
Education, n (%):							
More than a high school		23 (71.9)		77 (69.4)		41 (69.5)	
High school or less		9 (28.1)		29 (26.1)		15 (25.4)	
Other		0 (0)		4 (3.6)		2 (1.8)	
Not provided		0 (0)		1 (0.9)		1 (16.9)	
^a^^,^^b^Ethnicity/race, n(%)							
Asian or Pacific Islander		2 (6.2)		7 (6.3)		8 (10.7)	
Black/African American		1 (3.1)		3 (2.7)		0 (0)	
Caucasian/White		3 (9.4)		18 (16.2)		6 (8)	
European		13 (40.6)		28 (25.2)		28 (37.3)	
Hispanic		0 (0)		0 (0)		1 (1.3)	
Indigenous		1 (3.1)		6 (5.4)		2 (2.7)	
Middle Eastern		1 (3.1)		2 (1.8)		0 (0)	
North American		9 (28.1)		41 (36.9)		22 (29.3)	
South American		1 (3.1)		0 (0)		0 (0)	

**Note:** The CAM-ICU-7 and ICDSC are positive for delirium when their scores are 3–7 and 4–8, respectively. A state between normal cognitive function and delirium wherein a patient has less than clinical threshold symptoms of delirium (commonly referred to as subsyndromal delirium) corresponds to a CAM-ICU-7 or ICDSC score of 1–2 or 1–3, respectively. **Abbreviations:** APACHE-II, Acute Physiology and Chronic Health Evaluation II; CAM-ICU-7, Confusion Assessment Method Intensive Care Unit-7; ICDSC, Intensive Care Delirium Screening Checklist; IQR, interquartile range; SD, standard deviation; SSD, subsyndromal Delirium; SOFA, Sequential Organ Failure Assessment.

*Sex and gender were recorded for each participant. The proportion for female sex and gender are the same, as such only sex is displayed in the table.

^a^Fifteen missing patient ethnicity/race.

^b^The demographic questionnaire included an open-ended question (“If comfortable, please identify the ethnicity/ethnicities that you identify with in the space below”), which lead to the variability in race and ethnicity.

Patients were evaluated for delirium daily, for a total of 641 paired CAM-ICU-7/RASS and ICDSC assessments on 218 patients. When evaluated by the CAM-ICU-7, 30.3% (95%CI: 24.5–36.7) of patients had a CAM-ICU-7 score of 1–2, 28.0% (95%CI: 22.4–34.4) had a CAM-ICU-7 score of 3–5 and 18.3% (95%CI: 13.7–24.1) had a CAM-ICU-7 score of 6–7. When evaluated by the ICDSC, 50.9% (95%CI: 44.3–57.6) of patients had an ICDSC score of 1–3, 27.1% (21.5–33.4) and 34.4% (95% CI: 28.4–41.0) a score of 4–8. When considering the possible range of delirium scores (i.e., CAM-ICU-7 scores 0–7 and ICDSC scores 0–8), the CAM-ICU-7 and ICDSC scores had significant positive correlation (Pearson correlation coefficient r = 0.58, p<0.001). Overall agreement between CAM-ICU-7 and ICDSC as a measurement of delirium (i.e., CAM-ICU-7 score of 3–7 and ICDSC score of 4 or greater) was moderate (kappa = 0.51) (Table 2). However, the agreement between the two tools when less than clinical threshold symptoms of delirium were present (i.e., CAM-ICU-7 score 1–2 and ICDSC score 1–3) was only fair (kappa = 0.21). The most common ICDSC items identified in patients with less than clinical threshold symptoms of delirium are shown in Table 3.

**Table 2:** Correlation between CAM-ICU-7 and ICDSC as delirium measures.

ICDSC, n(%)	CAM-ICU-7, n(%)			
All Patients (n = 218)	Positive (n = 117)	Negative (n = 101)	Total	Kappa
Positive (n = 75)	62 (28.4)	13 (6.0)	75 (34.4)	
Negative (n = 143)	39 (17.9)	104 (47.4)	143 (65.6)	
Total	101 (46.3)	117 (53.7)	218 (100)	0.51

**Table 3:** Frequency of ICDSC items reported in patients with less than clinical threshold symptoms of delirium (n = 292).

**ICDSC item (n = 292)**	**n (%)**
Sleep wake cycle	157 (53.8)
Psychomotor agitation or retardation	83 (28.4)
Fluctuations	61 (20.9)
Disorientation	60 (20.5)
Altered level of consciousness	58 (19.9)
Inattention	53 (18.2)
Inappropriate mood or speech	21 (7.2)
Hallucination, delusion or psychosis	11 (3.8)
**CAM-ICU item (n = 205)**	**n (%)**
Acute onset or fluctuations	168 (81.9)
Inattention	19 (9.3)
Altered level of consciousness	71 (34.6)
Disorganized thinking	27 (13.2)

### Length of ICU stay

**Fig 3:**
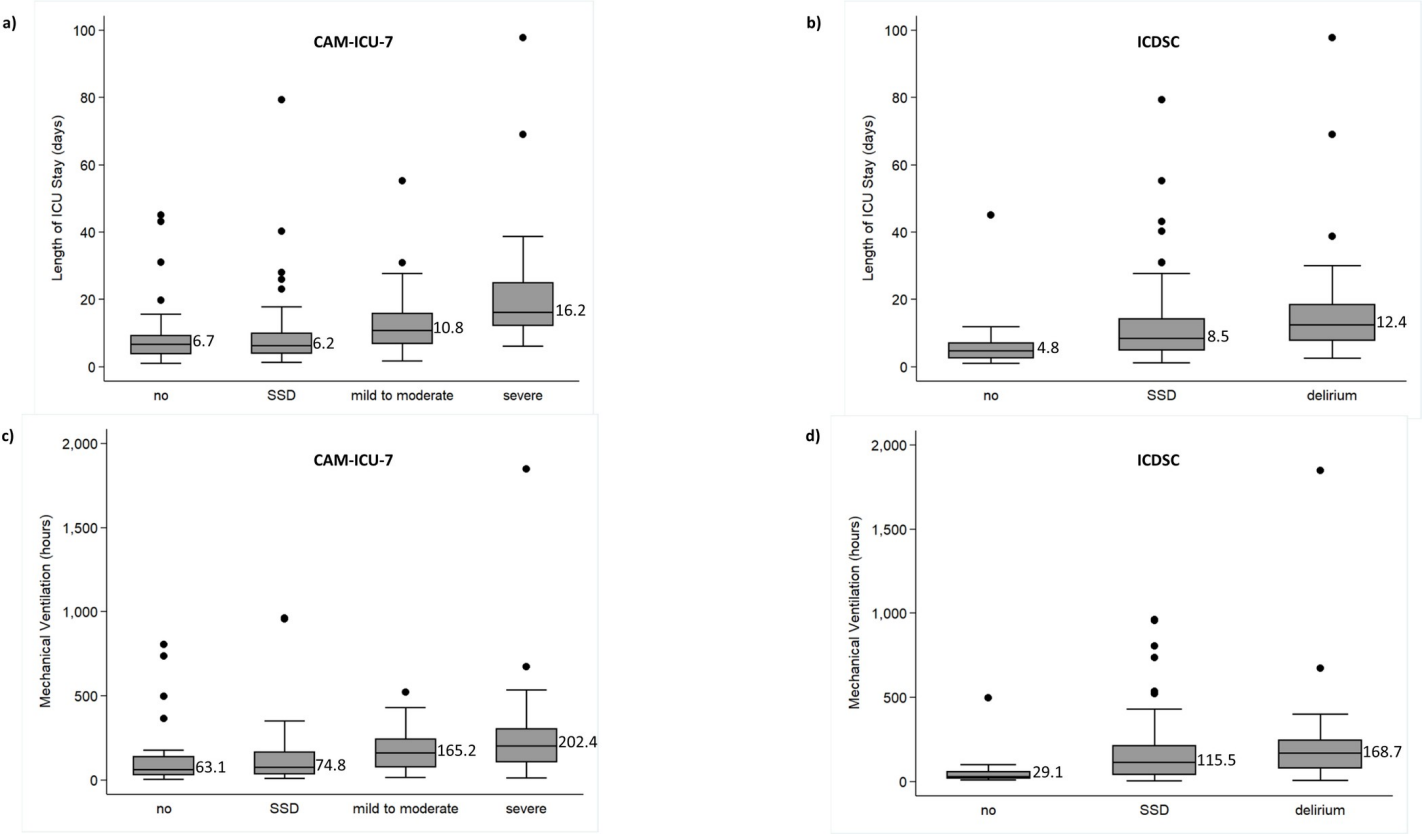
Median length of mechanical ventilation by type of delirium measured by the a) CAM-ICU-7 and b) ICDSC. Boxes indicate the first quartile, median (labeled) and third quartile. Circles indicate the outliers. One extreme outlier (3,399 hours) for mechanical ventilation is not shown. Median length of ICU stay by type of delirium measured by the c) CAM-ICU-7 and d) ICDSC. Boxes indicate the first quartile, median (labeled) and third quartile. Circles indicate the outliers. One extreme outlier (237 days) is not shown.

Tables 4 and 5 include the unadjusted (crude), stratified and adjusted odds ratios (ORs) of length of ICU stay associated with CAM-ICU-7 or ICDSC scores, respectively. There was no effect modification or confounding by patient sex, age or APACHE-II score for the CAM-ICU-7. Length of ICU stay was significantly associated with CAM-ICU-7 scores of 3–5 (crude OR 3.7, 95%CI: 1.5–7.5) and 6–7 (crude OR 51.4, 95%CI: 6.5–408.8), compared to patients who did not have delirium during their ICU stay (CAM-ICU-7 score of 0). There was no effect modification or confounding by patient sex or APACHE-II score for the ICDSC. There was effect modification by patient age (p = 0.007) for patients with an ICDSC score of 1–3: the odds ratio for length of ICU stay for patients <65 years of age (n = 76) was 9.2 (95% CI: 2.5–34.0) and for ≥65 years of age (n = 35) was 0.7 (95% CI: 0.2–2.7), when compared to patients who did not have delirium during their ICU stay (ICDSC score of 0). There was no effect modification or confounding for ICDSC score of 4 to 8 (crude OR 11.1, 95%CI: 5.2–29.2). There appeared to be a similar association between CAM-ICU-7 or ICDSC score and odds of prolonged length of ICU stay wherein an increased score on either tool was associated with increased median length of ICU stay (Fig 3).

**Table 4:** Unadjusted, adjusted and stratified odds ratio estimates associations between prolonged length of ICU stay (≥7 days) or mechanical ventilation (ever/never, ≥96 hours) and CAM-ICU-7 score.

	Unadjusted	Stratified*	Adjusted
**Length of ICU stay**	**OR (95% CI)**	**OR (95% CI)**	**OR (95% CI)**
CAM-ICU-7 score 0	1 [Reference]	_	1 [Reference]
CAM-ICU-7 score 1–2	0.9 (0.4–1.9)	NS	NS
CAM-ICU-7 score 3–5	3.4 (1.6–7.5)	NS	NS
CAM-ICU-7 score 6–7	51.4 (6.5–403.7)	NS	NS
**Mechanical Ventilation**	**OR (95% CI)**	**OR (95% CI)**	**OR (95% CI)**
CAM-ICU-7 score 0	1 [Reference]	_	1 [Reference]
CAM-ICU-7 score 1–2	1.4 (0.6–2.9)	<65: 0.5 (0.2–1.4)	_
		≥65: 9.6 (2.1–43.6)	
CAM-ICU-7 score 3–5	1.5 (0.7–3.4)	Female: 0.5 (0.1–1.9)	_
		Male: 3.0 (1.0–8.7)	
		<65: 0.8 (0.2–2.4)	
		≥65: 5.1 (1.2–22.3)	
CAM-ICU-7 score 6–7	10.4 (2.2–48.0)	**-**	12.5 (2.6–59.3)
**Mechanical Ventilation ≥96 hours**			
CAM-ICU-7 score 0	1 [Reference]	_	1 [Reference]
CAM-ICU-7 score 1–2	1.6 (0.7–3.7)	NS	NS
CAM-ICU-7 score 3–5	4.0 (1.7–9.3)	NS	NS
CAM-ICU-7 score 6–7	10.9 (4.1–29.0)	NS	12.8 (4.7–35.3)

*If there is effect modification, the odds ratio (OR) for the strata where there is significant interaction is reported. If there is no significant interaction or confounding with sex, age or APACHE-II score identified, the interaction is listed as not significant (NS).

**Table 5:** Unadjusted, adjusted and stratified odds ratio estimates associations between prolonged length of ICU stay (≥7 days) or mechanical ventilation (ever/ never) and ICDSC score.

	Unadjusted	Stratified	Adjusted
**Length of ICU stay**	**OR (95% CI)**	**OR (95%)**	**OR (95%)**
ICDSC score 0	1 [Reference]	_	1 [Reference]
ICDSC score 1–3	3.2 (1.4–7.6)	<65: 9.2 (2.5–34)	_
		≥65: 0.70 (0.2–2.7)	
ICDSC score 4–8	11.1 (4.2–29.2)	NS	_
**Mechanical ventilation**	**OR (95% CI)**	**OR (95%)**	**OR (95%)**
ICDSC score 0	1 [Reference]	_	1 [Reference]
ICDSC score 1–3	2.8 (1.3–6.4)	NS	NS
ICDSC score 4–8	6.5 (2.5–17.0)	NS	NS
**Mechanical ventilation ≥96 hours**			
ICDSC score 0	1 [Reference]	_	_
ICDSC score 1–3	6.6 (1.9–22.9)	NS	NS
ICDSC score 4–8	14.5 (4.0–51.9)	NS	NS

*If there is effect modification, the odds ratio (OR) for the strata where there is significant interaction is reported. If there is no significant interaction or confounding with sex, age or APACHE-II score identified, the interaction is listed as not significant (NS).

### Mechanical ventilation

A total of 74.8% of patients (163/218) were mechanically ventilated at any point during their ICU stay. Of these patients, 57.1% (93/163) were mechanically ventilated for ≥96 hours. Tables 4 and 5 include the unadjusted, stratified and adjusted ORs of receipt mechanical ventilation associated with CAM-ICU-7 and ICDSC scores, respectively. There was effect modification by patient age for receipt of mechanical ventilation in patients with a CAM-ICU-7 score of 1–2 (p = 0.001, patients <65 years of age [n = 45] was OR 0.5 [95% 0.2–1.4] and patients ≥65 years of age [n = 21] was OR 9.6 [95% CI: 2.1–43.6]), when compared to patients with no delirium (CAM-ICU-7 score of 0). A similar effect was observed with a CAM-ICU-7 score of 3–5 (p = 0.044, patients <65 years of age [n = 42] was OR 0.8 [95% 0.2–2.4] and patients ≥65 years of age [n = 19] was OR 5.1 [95% CI: 1.2–22.3]), when compared to patients with no delirium (CAM-ICU-7 score of 0). There was effect modification by patient sex for receipt of mechanical ventilation in patients with a CAM-ICU-7 score of 3–5 (p = 0.041, males [n = 34] was 3.0 (95% CI: 1.0–8.7) and females [n = 11] was 0.5 (95%CI: 0.1–1.9), compared to patients with no delirium (CAM-ICU-7 score of 0). There was confounding for CAM-ICU-7 score of 6–7 by patient age for receipt of mechanical ventilation (crude OR 10.4, 95%CI: 2.2–48.0; adjusted OR 12.5, 95% CI: 2.6–59.3) and mechanical ventilation for ≥96 hours (crude OR 10.9, 95%CI: 4.1–29.0; adjusted OR 12.8, 95% CI: 4.7–35.3). No effect modification or confounding was observed for any variable on the ICDSC. Receipt of mechanical ventilation was significantly associated with ICDSC scores of 1–3 (crude OR 2.8, 95%CI: 1.3–6.4) and 4–8 (crude OR 6.5, 95%CI: 2.5–17.0), compared to patients who did not have delirium during their ICU stay (ICDSC score of 0). Mechanically ventilation for ≥96 hours was significantly associated with ICDSC scores of 1–3 (crude OR 6.6, 95%CI: 1.9–22.9) and 4–8 (crude OR 14.5, 95%CI: 4.0–51.9), compared to patients who did not have delirium during their ICU stay (ICDSC score of 0). There appeared to be a similar association between length of mechanical ventilation and CAM-ICU-7 score or ICDSC score wherein an increased score on either tool was associated with increased median length of mechanical ventilation (Fig 3).

### Mortality

Due to the small number of patients who died in the ICU (n = 7), there were insufficient observations to calculate associations between mortality and CAM-ICU-7 or ICDSC scores.

## Discussion

We explored the relationship between the CAM-ICU-7 and ICDSC as measures of the spectrum of delirium severity in critically ill adults. The results of current study suggest the CAM-ICU-7 and ICDSC are significantly correlated as measures of the spectrum of delirium severity. In addition, CAM-ICU-7 and ICDSC scores have a dose-dependent association with prolonged length of ICU stay and mechanical ventilation ≥96 hours. The association between adverse clinical outcomes differs between the CAM-ICU-7 and ICDSC. For example, less than clinical threshold symptoms of delirium was significantly associated with prolonged length of ICU stay and mechanical ventilation in the ICDSC (not CAM-ICU-7).

The agreement between CAM-ICU-7 and ICDSC for a clinical delirium diagnosis has been reported in several studies [7–9,11]. In the current study, the kappa was moderate for agreement between the scales for measurement of overall delirium, which is similar to the kappa coefficients (0.50, 0.55) previously reported [9,10], suggesting these tools performed similarly across studies. However, when comparing the ability of these tools to identify less than clinical threshold symptoms of delirium, the agreement for these tools was only fair. When comparing these two tools, different characteristics may explain the variability in prevalence of patients with less than clinical threshold symptoms of delirium (i.e., 66 patients with a CAM-ICU-7 score of 1–2 versus 111 patients with an ICDSC score of 1–3). The CAM-ICU-7 is a point in time assessment of the patient’s mentation, in which the assessor asks the patient a series of cognitive questions (3-minutes), two different times during a 12-hour period. In contrast, the ICDSC score is calculated from the bedside registered nurse’s observations of the patient during a 12-hour shift, which may identify more symptoms of delirium (e.g., psychomotor agitation or retardation, inappropriate mood or speech, sleep wake cycle and fluctuations which are observed throughout the shift) and thus more patients with an ICDSC score of 1–3. Another key difference is that the tools measure different delirium features. For example, only the ICDSC measures a disturbance in the patient’s sleep-wake cycle. Patients admitted to the ICU often experience sleep disturbance [31]. As such, it is expected that many patients would score at least a one on the ICDSC (i.e., less than clinical threshold symptoms of delirium). In the current study, an ICDSC score of one due to sleep-wake cycle disturbance occurred in 21.6% (63/292) of ICDSC assessments that identified less than clinical threshold symptoms of delirium. Given the different operating characteristics and measurement of delirium features in the CAM-ICU-7 and ICDSC, similar scores on these tools may not result in similar delirium presentations in each patient.

The results of the current study have applicability to the most frequently used measures of delirium in critically ill adult patients (CAM-ICU-7 or ICDSC) in clinical practice. In the current study, less than clinical threshold symptoms of delirium are associated with increased length of ICU stay, receipt of mechanical ventilation and mechanical ventilation ≥96 hours in the ICDSC (not CAM-ICU-7). As such, there is a prognostic significance of less than clinical threshold of delirium symptoms (ICDSC score 1–3) wherein ICU staff should address these symptoms of delirium (with nonpharmacological strategies included in the most recent clinical practice guidelines) to reduce the ICDSC score to 0 (i.e., no delirium) and prevent these adverse clinical events from occurring [32]. These strategies could include targeting the most common ICDSC items identified in patients with less than clinical threshold symptoms of delirium: psychomotor agitation or retardation (pharmacologic and non-pharmacologic treatment of pain and agitation), disorientation (reorientation protocols), sleep wake cycle (assist-control ventilation, noise/light reduction) [33].

### Strengths and limitations

The current study had several strengths. The study sample was diverse, including critically ill patients with a wide range of critical illness diagnoses from a large, tertiary care medical centre. We collected twice-daily delirium assessments conducted by trained research assistants and by bedside RNs who are regularly audited for delirium assessments as part of an ICU delirium sustainability initiative. The study was co-designed with patients, researchers and clinicians and was registered *a priori*. The study protocol was tested during a pilot study [34]. Limitations include the cross-sectional nature of data collection, which resulted in a mixture of incident and prevalent delirium cases with no ability to distinguish. Small sample size led to large confidence intervals in subgroups and the inability to analyse the association between delirium score and mortality. Moreover, mortality in the study population is lower than the 9% reported by the Canadian Institute for Health Information [34], which is attributed to recruitment of a patient population with lower morality (less severe illness severity given requirements to be responsive to delirium assessments). The two screening tools were administered by two different raters (CAM-ICU was administered by trained research assistants while the ICDSC required a trained clinician). The inclusion of clinical judgement with the ICDSC assessment may have accounted for differences in delirium detection. The type and dose of sedative agents, antipsychotics or other medications that may have confounded a delirium diagnosis were not measured. The presence of a pre-existing neurologic illness such as dementia was not recorded. There was no difference between median days of delirium by delirium severity for the CAM-ICU and ICDSC. Future studies should explore the association between duration of severity of delirium and patient outcomes.

## Conclusions

The current study demonstrated that the CAM-ICU and ICDSC correlate in identification of the spectrum of delirium severity of critically ill adults. The CAM-ICU-7 and ICDSC have fair agreement for identifying less than clinical threshold symptoms of delirium and moderate agreement for identifying delirium. The data suggest that, as the CAM-ICU-7 and ICDSC scores increase, the odds of poor short-term outcomes (length of ICU stay and receipt and length of mechanical ventilation) increase.
